# Comparison of immunity-boosting regimens for COVID-19 upon initiation of immunosuppressive therapy (CIRCUIT): study protocol for a randomised, controlled clinical trial

**DOI:** 10.1136/bmjopen-2025-115259

**Published:** 2026-04-24

**Authors:** Dianne L Carey, Golo Ahlenstiel, Fabienne Brilot, David A Brown, Rowena Bull, Helen Crowther, Anthony Cunningham, Miles P Davenport, Peter Diamond, Nada Hamad, Amanda Johnston, Anthony D Kelleher, Frederick J Lee, Gail V Matthews, Kathy Petoumenos, Gesalit C Quichua, Sanjay Swaminathan, Judith Trotman, Stuart Turville, Steve Vukic, Peter Wong, Sarah C Sasson

**Affiliations:** 1 The Kirby Institute, University of New South Wales, Sydney, New South Wales, Australia; 2 Department of Gastroenterology, Blacktown and Mount Druitt Hospital, Sydney, New South Wales, Australia; 3 Western Sydney University, Sydney, New South Wales, Australia; 4 Kids Research, The Sydney Children’s Hospitals Network, Sydney, New South Wales, Australia; 5 The University of Sydney, Sydney, New South Wales, Australia; 6 New South Wales Health Pathology, ICPMR, Sydney, New South Wales, Australia; 7 University of New South Wales, Sydney, New South Wales, Australia; 8 Department of Clinical Immunology and Immunopathology, Westmead Hospital, Sydney, New South Wales, Australia; 9 School of Biomedical Sciences, University of New South Wales, Sydney, New South Wales, Australia; 10 Department of Haematology, Blacktown and Mount Druitt Hospital, Blacktown, New South Wales, Australia; 11 Western Sydney University, Penrith, New South Wales, Australia; 12 Westmead Institute for Medical Research, Sydney, New South Wales, Australia; 13 Leukaemia Foundation, Brisbane, Queensland, Australia; 14 Department of Haematology, St Vincent’s Hospital Sydney, Darlinghurst, New South Wales, Australia; 15 The University of Notre Dame Australia, Perth, Western Australia, Australia; 16 Department of Haematology, Westmead Hospital, Sydney, New South Wales, Australia; 17 Department of Clinical Immunology, St Vincent’s Hospital Sydney, Sydney, New South Wales, Australia; 18 New South Wales Health Pathology, Newcastle, New South Wales, Australia; 19 Department of Infectious Diseases, St Vincent’s Hospital Sydney, Sydney, New South Wales, Australia; 20 Department of Haematology, Concord Hospital, Sydney, New South Wales, Australia; 21 Brain and Nerve Research Centre, The University of Sydney, Sydney, New South Wales, Australia; 22 Department of Neurology, Concord Hospital, Sydney, New South Wales, Australia; 23 Departments of Rheumatology Blacktown and Westmead Hospitals and Westmead Clinical School, The University of Sydney Faculty of Medicine and Health, Sydney, New South Wales, Australia; 24 New South Wales Health Pathology, ICPMR, Wentworthville, New South Wales, Australia

**Keywords:** COVID-19, Vaccination, Immunity, SARS-CoV-2 Infection

## Abstract

**Introduction:**

Immunosuppression is associated with an increased risk of delayed SARS-CoV-2 viral clearance, severe COVID-19 and related death. This heterogeneous group of affected patients includes but is not limited to those with a haematological malignancy, people on immunosuppressive therapy for the treatment of autoimmune/inflammatory diseases and those following bone marrow transplantation (BMT). Immunosuppression is associated with decreased rates of anti-spike IgG seroconversion following COVID-19 vaccination. While clinical guidelines have been established to guide vaccination pre-splenectomy and post-BMT, there are limited data to guide timing of COVID-19 or other booster vaccines in adults commencing new or intensified moderate to severe immunosuppression. The comparison of immunity-boosting regimens for COVID-19 upon initiation of immunosuppressive therapy (CIRCUIT) study was designed to address this knowledge gap. CIRCUIT investigates whether administration of a third (or subsequent) COVID-19 booster vaccine ≤2 weeks prior to immunosuppression provides greater anti-spike IgG-mediated immunity than a booster given 24 weeks after new or intensified immunosuppression, that is, week 24 timepoint (Group 1; n=280). Additionally, the research will investigate whether giving a fourth post-BMT COVID-19 booster vaccine at 9 months post-transplant provides greater anti-spike IgG-mediated immunity than a booster given 15 months post-transplant (Group 2; n=40).

**Methods and analysis:**

The CIRCUIT study is an open-label, multicentre randomised clinical trial. Participants will be randomised 1:1 to receive either an additional COVID-19 booster ≤2 weeks pre-immunosuppression and a diphtheria/tetanus toxoids (DT) booster at 24 weeks following new or intensified immunosuppression (week 24 timepoint) or receive a DT booster ≤2 weeks pre-immunosuppression and an additional COVID-19 booster at week 24 (Group 1). Group 2 participants who underwent autologous or allogenic BMT in the last 9 months will be randomised 1:1 to either receive a fourth post-BMT COVID-19 booster at 9 or 15 months post-transplant. The primary outcome will be the integrated time-weighted area under the curve anti-SARS-CoV-2 neutralising antibody (NAb) response over 12 months from a SARS-CoV-2 booster as assessed by a high-throughput SARS-CoV-2 NAb platform assay. Key secondary outcomes of the CIRCUIT randomised control trial will include safety and generation of SARS-CoV-2 antigen specific T and B cell responses.

**Ethics and dissemination:**

The research protocol was approved by the Western Sydney Local Health District Human Research Ethics Committee on 25 August 2022 (Ref no. 2022/PID00782 – 20022/ETH0069). Study results will be published in peer-reviewed medical journals and presented at local and international conferences. All findings regardless of the outcome will be reported.

**Trial registration number:**

NCT05415267.

**STRENGTHS AND LIMITATIONS OF THIS STUDY**

- For the first time, a randomised clinical trial will be used to measure differences in seroconversion to a COVID-19 booster given at baseline versus at 24 weeks in adults commencing new or intensified moderate to severe immunosuppression.
- This study includes a variety of immunosuppressed adults including those with a haematological malignancy and a range of autoimmune/inflammatory diseases encompassing high burden diseases such as inflammatory bowel disease and multiple sclerosis as well as rarer conditions, sub-stratified by treatment type.
- The study has statistical power to analyse Group 1 participants as a whole or by distinct substrata-based type of immunosuppression.
- For pragmatic reasons not all types of conditions associated with immunosuppression are included in the study; exceptions include those with solid organ malignancy, renal transplant recipients, HIV-infected individuals and those with primary immunodeficiencies.
- In Australia, COVID-19 vaccines can only be administered in state-government mandated centres not by hospital study staff therefore blinding of participants and research staff as to which vaccine was given was not possible and is a limitation of the study.

## Introduction

### Background and rationale

 It was recognised early in the COVID-19 pandemic that immunocompromised individuals were at increased risk of severe disease,^1 2^ delayed viral clearance^3 4^ and death^5 6^ compared with the general population. Immunocompromised individuals comprise heterogenous groups including, but not limited to, those with solid organ and haematological malignancies, those with autoimmune and inflammatory disease on immunosuppressive therapies, allogenic and autologous bone marrow transplantation (BMT) recipients and solid organ transplant recipients. While these groups were prioritised in many COVID-19 vaccine programmes, evidence soon followed that vaccine seroconversion, including titres of anti-SARS-CoV-2 spike protein IgG were lower in immunocompromised individuals compared with healthy comparators. Patients with follicular lymphoma treated with immunochemotherapy had lower anti-spike IgG following two doses of BNT162b2 vaccine compared with those who were untreated and to healthy controls.^7^ Similarly, in patients with Waldenström Macroglobulinemia, those receiving Bruton’s tyrosine kinase inhibitors (BTKis) had lower anti-spike IgG following two doses of BNT162b2 vaccine compared with untreated counterparts and healthy controls.^7^ Studies of a third dose of COVID-19 vaccine in patients with haematological malignancies have demonstrated heterogeneous improvement in anti-spike IgG levels dependent on disease state and therapy. A retrospective observational study of 381 patients with haematological malignancies who received a third dose of a mRNA COVID-19 vaccine found detectable anti-spike IgG in 73.1% of patients after two vaccine doses which rose to 86.4% after three doses.^8^ Rates of seroconversion were lower in patients with chronic lymphocytic leukaemia (CLL) (52.6%) and B cell lymphoma (60.9%).^8^ While the third vaccine dose improved seroconversion for patients on Janus kinase inhibitors (JAKi) (from 57.1% to 90.9%), the rates of seroconversion remained low for patients with haematological malignancies on anti-CD20 treatment (55%), the bcl-2 inhibitor venetoclax (50%) and BTKis (37.5%).^8^ An additional study of 172 patients with CLL/small lymphocytic lymphoma (SLL) who did not seroconvert after two doses of a BNT162b2 vaccine received a third dose of the same vaccine.^9^ Overall, 23.8% of patients with CLL/SLL seroconverted following the third BNT162b2 vaccine with the highest rates among patients who were treatment naïve (40.6%) or off treatment (40%) while lower seroconversion was observed among those on active treatment (12%).^9^ Only one of 28 patients who had received an anti-CD20 B-cell depletion agent in the preceding year seroconverted (3.6%).^9^

B-cell depletion therapies and sphingosine-1-phosphate receptor 1 inhibitors that inhibit lymphocyte egress from lymph nodes have consistently demonstrated detrimental effects on COVID-19 vaccine seroconversion including in the setting of multiple sclerosis^10^ and immune mediated inflammatory diseases.^11^ In patients with inflammatory bowel disease, those treated with tumour necrosis factor-alpha (TNFα) inhibitors had reduced seroconversion to the first two doses of a COVID-19 vaccine compared with those treated with the gut-specific alpha 4 beta 7 (α4β7)-integrin inhibitor vedolizumab.^12^ Autoimmune and inflammatory conditions include both rare and more common diseases and are challenging to study due to the clinical heterogeneity of the underlying conditions and the use of non-protocolised treatment regimens that include both conventional synthetic immunosuppressive agents as well as more targeted immunomodulator therapies. A study conducted in patients with immune-mediated inflammatory diseases receiving their first two doses of BNT162b2 vaccine found median anti-spike IgG levels were lower than in healthy controls at 28 days. Lower median seroconversion responses were also observed in patients receiving both conventional synthetic disease-modifying agents (eg, hydroxychloroquine, methotrexate and sulfasalazine) and targeted biologic therapies eg, anti-interleukin (IL)-6, IL-17, IL-23, TNFα and JAKi.^13^ However, post COVID-19 vaccination serologic responses have not been stratified according to the type of immunosuppression. There is some evidence that patients with autoimmune disease on methotrexate-containing regimens have lower seroconversion responses following COVID-19 vaccinations compared with those not taking methotrexate-containing regimens.^14^ A prospective, open-label randomised control trial which studied 804 immunosuppressed patients, including those with autoimmune/inflammatory disorders, found a suboptimal response following two doses of a COVID-19 vaccine.^15^ 90% of patients categorised as ‘low-responders’ after two doses increased their anti-spike IgG antibodies to the median of the general population, however, 54% of patients categorised as ‘non-responders’ again had no detectable seroconversion after the third dose, suggesting that in some patients a suboptimal serological response cannot always be overcome by administering additional doses.^15^ Again, patients receiving rituximab and other B-cell depletion agents had anti-spike IgG levels markedly below the median of the general population.^15^ Patients treated with corticosteroids and calcineurin inhibitors had mildly reduced seroconversion while those treated with antimetabolites, conventional disease-modifying agents and biologics were less affected.

There are limited data to guide timing of booster vaccine administration around the commencement of moderate-to-severe immunosuppression. Current protocolised clinical guidelines for vaccination in the setting of immunodeficiency predominantly relate to two specific scenarios, those of splenectomy and BMT.

In planned or non-emergency splenectomy, vaccination is recommended at least 2 weeks prior to surgery to prevent overwhelming post-splenectomy infection particularly related to encapsulated bacteria.^16^ The pre-splenectomy schedule includes vaccination to Pneumococcus, meningococcal ACWY, meningococcal B, Haemophilus influenza type B (Hib) with booster doses to Pneumococcus, meningococcal ACWY and meningococcal B given at 2 months and 5 years in addition to a seasonal influenza vaccine.

Vaccination guidelines for adults who have undergone autologous or allogenic BMT advise a comprehensive re-vaccination programme commencing 6 months post-BMT.^16^ Revaccination is required for diphtheria/tetanus (DT; three doses total), Hib (three doses, 4 weeks apart), hepatitis B (three doses at 6, 8 and 12 months post-BMT), pneumococcus (three doses of a conjugate vaccine 4 weeks apart followed by two doses of a 23-valent vaccine), inactivated poliovirus (three doses, 4 weeks apart), MenACWY (two doses, 8 weeks apart), meningococcal B (two doses) and herpes zoster (two doses, 2–6 months apart) with an annual seasonal influenza vaccine also recommended. Additional vaccines given at 24 months post-BMT at the discretion of the treating physician include measles mumps and rubella (two doses, 4 weeks apart) and varicella zoster (two doses, 4 weeks apart) provided there is no evidence of graft-versus-host-disease, and immunosuppressive therapy has been ceased for more than 3 months.

Prior to the COVID-19 pandemic there were limited evidence-based national or international guidelines around the timing of adult booster vaccinations for those with moderate to severe immunosuppression apart from splenectomy and BMT. In response to the COVID-19 pandemic, guidelines advising COVID-19 vaccinations including booster vaccines should be given at least 2 weeks prior to rituximab therapy emerged based on evidence of improved anti-spike IgG seroconversion in patients who had received COVID-19 vaccines 2–4 weeks prior to B-cell depletion.^17^,^19^ However, data relating to other forms of immunosuppression are lacking, and for this reason, the CIRCUIT study was conceived to determine the optimal timing of COVID-19 booster vaccines in two adult patient groups; Group 1: adults commencing new or intensified moderate-to-severe immunosuppression for the treatment of a haematological malignancy or an autoimmune/inflammatory disease and Group 2: adults who have undergone allogenic or autologous BMT in the previous 9 months. Due to a related study running concurrently in Australia,^20^ people with HIV infection and solid organ transplant recipients were not included in the CIRCUIT study.

### Explanation for choice of comparator

Group 1 participants will be randomised to receive either a baseline (week 0) COVID-19 booster prior to commencing new or intensified immunosuppressive therapy and a DT booster at week 24 (Arm A) or a baseline DT booster and a week 24 COVID-19 booster (Arm B).

Participants in Group 2 will have undergone a BMT in the previous 9 months and received three post-BMT COVID-19 vaccines. These participants will be randomised to either receive their fourth post-BMT COVID-19 vaccine at baseline (week 0) (Arm C) or at week 24 (Arm D).

Due to the evolving nature of COVID-19 vaccine development, the study will accept any COVID-19 vaccine licensed for use in Australia. Due to established guidelines on post-BMT vaccination including diphtheria and tetanus, only COVID-19 boosters will be studied in Group 2.^16^

The DT vaccine was selected as the comparator for the COVID-19 vaccine. The DT vaccine is protein-based and stimulates an immune response via a different mechanism to that of currently available mRNA-based COVID-19 vaccines. The study will assess whether the findings that relate to the optimal timing of the COVID-19 booster also apply to more traditional protein-based vaccines. If this is found to be the case, then the study results may have broader implications for vaccinology and optimal clinical guidelines.

### Objectives

This study aims to evaluate the optimal timing of a COVID-19 booster vaccination in adults commencing moderate-to-severe immunosuppression for either an autoimmune/inflammatory disease or a haematological malignancy or who have undergone a bone marrow transplant in the previous 9 months. Efficacy will be assessed primarily by measuring the anti-SARS-CoV-2 neutralising antibody (NAb) response to the booster over 12 months. Other correlates of protective immunity as well as safety and quality of life will be assessed.

Delaying administration of the COVID-19 booster in the deferred arms may lead to higher numbers of breakthrough COVID-19 infections in these arms. The Data Safety Management Board (DSMB) will closely monitor on-study infections and advise the principal investigator and Protocol Steering Committee (PSC) should there be any concerns.

## Methods and analysis

### Patient and public involvement

The study was developed with the support of key consumer organisations including Arthritis Australia, Crohn’s and Colitis Australia, The Leukaemia Foundation and MS Australia. A Leukaemia Foundation representative is a named co-investigator, contributing to the design and execution of the study and is a member of the PSC. Additionally, the study design was presented to The Kirby Institute UNSW’s COVID-19 Community Advisory Board which includes representatives from The Lung Foundation, Spleen Australia and the Transplantation Society of Australia and New Zealand. Following the completion of the study, key results and findings will be disseminated through these and other community stakeholder organisations.

### Trial design

CIRCUIT is an open-label, multicentre randomised clinical trial. Participants with a haematological malignancy or autoimmune/inflammatory disease will be randomised in a 1:1 ratio to receive either an additional COVID-19 booster pre-immunosuppression with a DT booster after 6 months of therapy or a DT booster pre-immunosuppression and an additional COVID-19 booster at 6 months. Participants at least 9 months post-BMT will be randomised in a 1:1 ratio to either receive a COVID-19 booster either following randomisation or after 6 months.

### Trial setting

Eligible participants will be recruited from five academic hospitals throughout Sydney, Australia.

### Eligibility criteria

Systemic autoimmunity and haemato-oncology group (Group 1)

#### Inclusion criteria

- Adult aged ≥18 years with a medical condition included in table 1.
- Previously vaccinated with at least two doses of any licensed COVID-19 vaccine who requires initiation of moderate to severe immunosuppression; the most recent vaccination must have been given more than 3 months prior.
- Planned significant immunosuppressive therapy for at least 1 year (table 1).
- No cyclophosphamide, alemtuzumab or rituximab treatment in the past 5 years (concurrent corticosteroids with treatments listed in table 1 are permitted).
- Voluntary written informed consent.

**Table 1:** Study populations

Group 1*	
**Haemato-oncology**	**Systemic autoimmunity**
Haematological malignancy (excluding bone marrow transplant recipients)† (n=40) B-CLL Follicular or marginal zone lymphoma Multiple myeloma Diffuse large B-cell lymphoma Other haemato-oncology condition at the discretion of the study PI or their delegate	ANCA-positive vasculitis,‡ ANCA-negative vasculitis,‡ Polyarteritis nodosa,‡ Churg-Strauss,‡ Ankylosing spondylitis,‡ Autoimmune hepatitis,§ IgG4 disease,‡ Inflammatory bowel disease,¶** Psoriatic arthritis,‡ Psoriasis, Rheumatoid arthritis,‡ SLE,‡ Sjogren’s syndrome,‡ Myasthenia gravis,†† Multiple sclerosis,‡‡ Autoimmune encephalitis, Neuromyelitis optica,¶ Bullous pemphigoid,¶ Pemphigus vulgaris¶ and variants, Sarcoidosis, Uveitis, other autoimmune/inflammatory conditions diagnosed by a specialist physician and at the discretion of the study PI or their delegate.
	B-cell depletion therapies, eg, rituximab, ocrelizumab, ofatumumab (n=40)
	Anti-metabolite therapies: azathioprine, calcineurin inhibitors, mycophenolate or methotrexate (moderate immunosuppression) (n=40)
	TNFα inhibition (with or without azathioprine or methotrexate or leflunomide to prevent anti-drug antibodies) (n=40)
	Cyclophosphamide or alemtuzumab (severe immunosuppression) (n=40)
	Janus kinase (JAK) inhibitors±leflunomide (n=40)
	Anti-IL-17 and/or 23 monoclonals, eg, secukinumab (n=40)

Group 2
*Bone marrow transplant (n=40)* Haematological malignancy—autologous or allogenic transplant (excluding for treatment of a primary immunodeficiency)

* Eligible patients with both a haematological malignancy and a systemic autoimmune condition are to be enrolled in the haematological malignancy sub-stratum.

† WHO criteria.

‡ EULAR criteria.

§ Biopsy proven.

¶ Mayo criteria.

** Crohn’s Disease activity index.

†† McDonnel I criteria.

‡‡ Must have AChR or Musk autoantibody positive.

AChR, Acetylcholine Receptor; ANCA, Antineutrophil Cytoplasmic Antibodies; EULAR, European Alliance of Associations for Rheumatology; IL, Interleukin; PI, Principal Investigator; TNF, tumour necrosis factor.

#### Exclusion criteria

- Pregnant or breastfeeding.
- Underlying primary immunodeficiency.
- Received or likely to receive intravenous/subcutaneous immunoglobulin (IVIg/SCIg).
- Projected treatment is likely to involve plasma exchange.
- Contraindication to receipt of a COVID-19 vaccine.
- Intolerance of or previous allergic reaction to tetanus vaccination.
- If switching immunosuppressive therapies following enrolment absolute neutrophil count must be above 0.5×10^9^/L at screening.

Bone marrow transplant group (Group 2)

#### Inclusion criteria

- Adult aged ≥18 years with a medical condition included in table 1.
- Undergone a BMT for a haematological malignancy in the previous 9 months.
- Previously received at least three post-BMT doses of any licensed COVID-19 vaccine as per current standard of care post-transplant guidelines.^16^
- Voluntary written informed consent.

#### Exclusion criteria

- Pregnant or breastfeeding.
- Underlying primary immunodeficiency.
- Received or likely to receive IVIg/SCIg.
- Projected treatment is likely to involve plasma exchange.
- Contraindication to receipt of a COVID-19 vaccine.

### Intervention and comparator

Only participants with a systemic autoimmune/inflammatory disease or a haemato-oncology condition (Group 1) will receive the DT intervention. Participants randomised to the deferred COVID-19 booster will receive the DT booster prior to starting immunosuppressive therapy and those randomised to the immediate COVID-19 booster will receive the DT booster at 6 months.

Although all participants will receive a COVID-19 booster, it is not a study intervention. It will be administered at vaccination sites in general practice sites and pharmacies and provided free of charge as part of the Australian Government National COVID-19 Vaccine Programme.

### Criteria for discontinuing or modifying allocated intervention

Study participation is voluntary, and participants can withdraw consent or cease participation at any time without penalty. Investigators may also withdraw participants for several reasons including progression of underlying disease; occurrence of unacceptable adverse events (AEs); continued participation is considered not to be in the participant’s best interest; or either party wishes to cease participation.

### Strategies to improve adherence to intervention protocols

Investigators and study coordinators will encourage participant attendance at follow-up visits to ensure data collection is maximised. Following randomisation participants will be provided with a schedule detailing dates of study visits. To reduce the number of on-site visits, the visit schedule permits screening and randomisation to be conducted on the same day. Additionally, participants randomised to the deferred COVID-19 booster can receive their DT booster at this time further reducing the need for a return visit. Study staff will ensure data on AEs, and health questionnaires are completed during study visits and will assist in ensuring questionnaire completion. As study visit attendance can incur expenses, participants will be reimbursed in the form of a $30 gift card for each on-site visit attendance.

### Concomitant care permitted or prohibited during the trial

During the trial participants will receive standard of care management for their study qualifying medical condition as well as other medical conditions that might arise. Participants should not receive additional COVID-19 boosters during the trial as it will alter assessment of the immune responses to the COVID-19 booster administered on-study. All doses of tixagevimab/cilgavimab (Evusheld) administered for COVID-19 prevention will be recorded in case report forms. For patients who receive tixagevimab/cilgavimab, the anti-spike IgG assays will be modified to discriminate between host-derived anti-spike IgG and the synthetic monoclonal antibodies. This can be achieved because tixagevimab/cilgavimab has been modified to not bind to Fc gamma receptors (FcγRs).^21^ The FcγR is an endogenous receptor that binds naturally occurring antibodies produced by the host. The modifications made to tixagevimab/cilgavimab were aimed at reducing antibody-dependent cellular cytotoxicity and related inflammation.^21^ Therefore, including a ‘positive selection’ FcγR binding step in the anti-spike IgG assay allows for the detection of host-produced anti-spike IgG and not the synthetic antibodies.

### Outcomes

The primary outcome will be the integrated time-weighted area under the curve (AUC) anti-SARS-CoV-2 NAb response over 12 months from a SARS-CoV-2 booster as assessed by a high-throughput SARS-CoV-2 NAb platform assay developed by our group.^22^,^24^ The assay involves culturing a cell-line that is hyper-permissible to SARS-CoV-2 through the expression of ACE2 and transmembrane serine protease 2 receptors. Serial dilutions of patient serum are then introduced to the cell culture wells in addition to homogenous concentrations of SARS-CoV-2 virions including variants of concern. Anti-spike IgG present in the patient serum acts to block viral entry into the cell. The assay readout is the infectious dose 50% (ID_50_), the viral concentration required to obtain 50% cellular death.

Secondary outcomes include laboratory, safety and clinical/biological outcomes:

Laboratory outcomes:

- NAb response to a tetanus toxoid booster integrated over 12 months.
- Responses to the COVID-19 vaccine including
  - Breadth of NAb against SARS-CoV-2 variants of concern.
  - Kinetics of NAb over time including responses to variants of concern.
  - Memory T cell, B cell and natural killer (NK) cell responses to peptide sets representing different domains of the SARS-CoV-2 spike protein.
- Immunological responses as assessed above in populations stratified by broad study-qualifying disease type and different modes of immunosuppression employed such as chemotherapy, BMT, B-cell depleting therapies and cytokine inhibition.

Safety outcomes:

- Local and systemic AEs that occur in the 7 days following the DT and COVID-19 vaccinations.
- AEs following immunisation and adverse drug reactions following immunisation for the duration of the study.
- COVID-19 vaccine-induced disease flares.
- On-study PCR-confirmed or rapid antigen-confirmed SARS-CoV-2 infections.
- All on-study pregnancies.

Clinical/biological outcomes:

- Health quality of life scores (EQ-5D-3L) at weeks 0, 24 and 48.
- Impact of immunosuppression on vaccine protection over time will be assessed by modelling:
  - Level of protection over time based on NAb levels.
  - Boosting and decay of anti-spike IgG, specific memory B, T and NK cell responses.
- Inform policies on the timing of additional COVID-19 boosters in vulnerable populations about to commence immunosuppression or following BMT.

### Harms

All AEs occurring in the 7 days following booster vaccinations will be recorded on the electronic case report form (eCRF). Events will be MedDRA coded at data entry and details of their duration, severity, relatedness to the vaccination and management will be collected. All AEs and serious adverse events (SAEs) not related to participants’ underlying medical condition that qualified them for the trial, or to its treatment will be recorded on the eCRF. SAEs will be monitored continuously throughout the trial and will be recorded on both the eCRF and separately for onward reporting to the sponsor and ethics committee within 24 hours of an investigator becoming aware.

### Participant timeline

**Figure 1:**
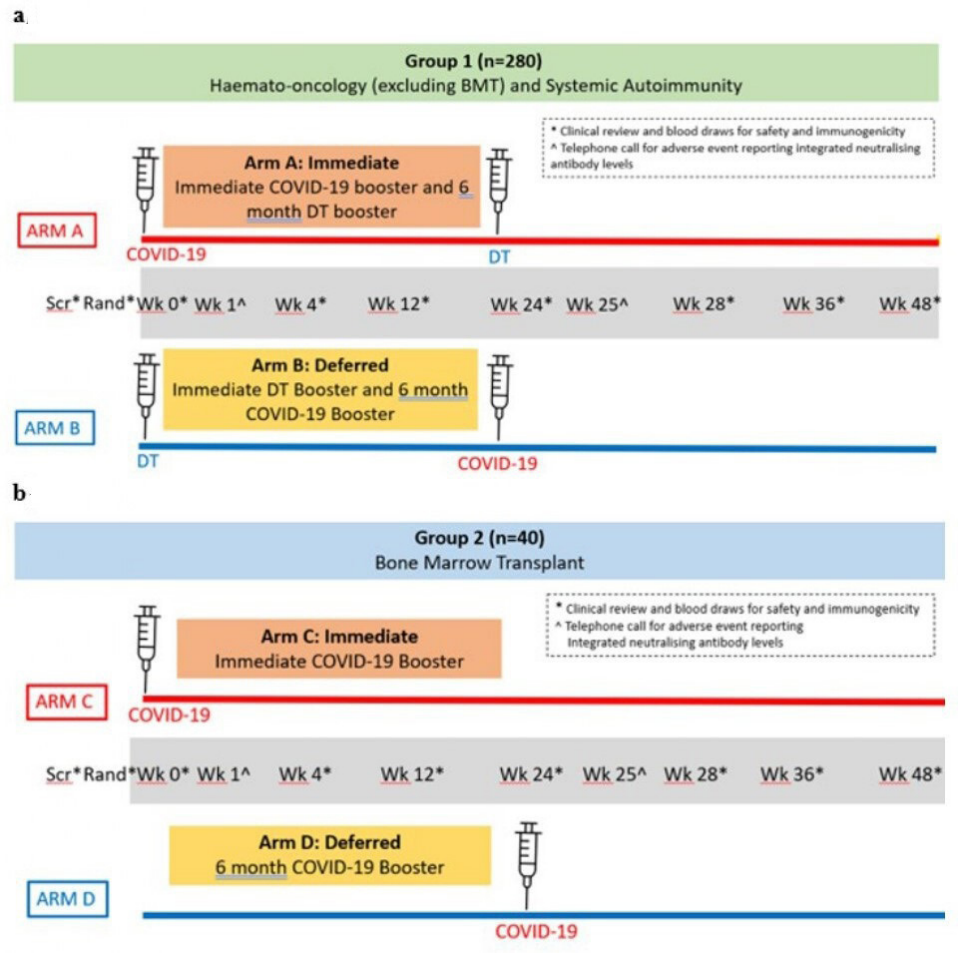
Trial design. (a) Group 1: haemato-oncology and systemic autoimmunity disease participants. (b) Group 2: bone marrow transplant recipients. BMT, bone marrow transplant; DT, diphtheria/tetanus toxoids; Rand, randomisation; Scr, screening; Wk, study week.

The study design and participant timeline are shown in figure 1. Participants in Group 1 will have up to 11 visits over 12 months and those in Group 2 a maximum of 10.

### Sample size

The maximum sample size is 320 participants, 280 in the systemic autoimmunity and haemato-oncology group and 40 in the BMT recipient group. This sample size is based on previously calculated integrated time-weighted AUC of live anti-SARS-CoV-2 NAb titres following two SARS-CoV-2 vaccinations in healthy adults^25^ and patients infected with COVID-19.^26 27^ SD calculated from these datasets were 0.11 log_10_ and 0.12 log_10_, respectively. Assuming an SD of 0.12 log_10_, the study will have over 90% power to detect a 0.13 log_10_ difference in time weighted AUC log_10_ change from baseline (2-alpha=5%) within each sub-stratum of 40 participants. This corresponds to a 35% difference in live virus neutralisation titre between randomised arms. Time-weighted AUC allows for varying of follow-up of participants due to missed visits, loss to follow-up or death. We conservatively estimate overall lost to follow-up to be 10% and mortality 1% balanced across randomised sub-strata.

### Recruitment

Eligible participants will be identified at participating sites by clinicians involved in the management of eligible patient groups in both inpatient and ambulatory settings. Approved promotional material will target eligible patient groups to enhance recruitment.

### Randomisation

#### Sequence generation

Participants will be assigned to a study group based on their study-qualifying medical condition (table 1) using a central computer-generated randomisation sequence with a blocking factor of four created by the study statistician.

Participants in Group 1 will be stratified into one of two supra-stratum, haematological malignancy or systemic autoimmunity and then to sub-strata based on their planned treatment. Sub-strata include: haematological malignancy—chemotherapy; autoimmunity—B-cell depletion therapies; autoimmunity—anti-metabolite therapies; autoimmunity—TNFα-inhibition; autoimmunity—cyclophosphamide or alemtuzumab; autoimmunity—JAKi; autoimmunity—anti IL-17/23 monoclonal antibodies (table 1).

Group 1 participants will be randomised in a 1:1 ratio to receive either a COVID-19 booster at week 0 and a DT booster at week 24 (Arm A: immediate booster), or a DT booster at week 0 and a COVID-19 booster at week 24 (Arm B: deferred booster) (figure 1). Randomisation will be conducted using a central computer-generated random allocation algorithm using blocks and stratified by sub-strata population.

Group 2 has no sub-strata and participants will be randomised in a 1:1 ratio to receive either an immediate COVID-19 booster at week 0 (Arm C), or a deferred COVID-19 booster at week 24 (Arm D) (figure 1).

### Allocation concealment mechanism

Following central computer randomisation, participants’ assignments to either immediate or deferred COVID-19 booster will appear on the screen. The randomisation screen will be printed and form part of participants’ study records. The screen print plus a prescription will be provided to an on-site clinical trials pharmacy for dispensing of the DT vaccine.

### Implementation

Members of the site’s research team will enrol participants who will be randomly assigned to the intervention as described. The DT booster will be administered at the site by a qualified member of the research team. The COVID-19 booster will be administered at local vaccination sites.

### Blinding

This study was implemented in Sydney, Australia during the pandemic phase of COVID-19. During that time, the New South Wales (NSW) Ministry of Health had control over the limited supply of COVID-19 vaccine. Hence, we were unable to secure a supply of vaccines for the study. Therefore, the trial was designed so that COVID-19 boosters could be given within the state government mandated vaccination hubs and centres. As there were no supply or administration restrictions for the DT booster, it could be given to participants at the hospital study sites by research staff. Therefore, due to the different administration locations for the COVID-19 and DT vaccines, it was not possible to blind either the participants or research staff.

### Data collection methods

Following enrolment, research coordinators will collect relevant demographics (age, gender, ethnicity) and clinical data (vital signs, physical measurements, study-qualifying disease diagnosis and treatment plan, medical history, pregnancy status, concomitant medication and COVID-19 infections and vaccinations) on a trial-specific eCRF. Following randomisation and initial booster vaccination, the visit schedule will include eight visits for participants in Group 1 and seven for those in Group 2.

The schedule of study assessments is outlined in online supplemental table 1. Blood samples will be collected at baseline and all on-site follow-up visits for assessment of immunological outcomes. At all study visits, a research coordinator will collect relevant clinical data on the visit eCRF and will review concomitant medication, AEs and SAEs that may have occurred since the last visit. Quality of life assessments at baseline and at weeks 24 and 48 will be self-reported using the validated EQ-5D-3L tool^28^ and participant responses will be entered into the eCRF by the coordinator.

### Plans to promote participant retention and complete follow-up

Each participant will be provided with a visit schedule outlining visit dates and the visit windows so attendance can be planned. Research coordinators will contact participants prior to visits to promote attendance. Regular newsletters will be sent to research sites to promote the study, encourage enrolment and provide updates on progress and retention. Refresher training video meetings will also be conducted if necessary. Completion of study data will be checked at regular intervals by the main study coordinator.

### Data management

Research data collected on eCRFs and study-specific forms will be stored in a secure password-protected, encrypted study-specific clinical trial management system (Research Electronic Data Capture (REDCap) hosted by the sponsor, UNSW Sydney. This electronic data capture (EDC) system contains all clinical data, AEs and questionnaire responses and is based on source documentation maintained at research sites. Trained research coordinators at sites will enter data into the EDC. Automated checks will ensure entered data conform to trial-specific procedures. Data cleaning and quality checks will be conducted on an ongoing basis by trained personnel to ensure data are accurate and complete. Additionally, on-site monitoring visits will be conducted during the trial to verify source documentation is accurate, complete, consistent and adheres to the study protocol. Data will be backed up regularly and only available to study researchers. Data will be stored for a minimum of 15 years following which the storage media will be physically destroyed.

### Statistical methods

#### Statistical methods for analysing primary and secondary outcomes

Baseline participant characteristics will be summarised by randomised arm for each sub-stratum separately and combined. Primary analyses will compare immunogenicity endpoints between randomised arms on an intention-to-treat basis, that is comparing participants as randomised regardless of whether they received immediate or deferred vaccinations according to the protocol. Log_10_ NAb titres will be summarised at each time point and presented graphically within each stratum. The primary outcome will be time-weighted AUC change from baseline in anti-SARS-CoV-2 log_10_ NAb titre over 12 months. The primary analysis will follow a modified intention to treat approach (mITT) and include all randomised participants with a baseline visit and at least one post baseline time point measured. This approach allows for the inclusion of all participants, even with partial follow-up. The AUC will be calculated for each participant using the trapezoidal rule based on all available data. To assess for bias due to missing data, a secondary analysis which will only include patients who completed the study and have no missing data will be performed. These results will be compared with the mITT analysis. Additionally, if the proportion of patients with missing data is over-represented in one study arm, additional sensitivity analyses will be performed. Randomised arms will be compared within each treatment stratum separately using simple, unadjusted two-sample t-tests. In the event of unbalanced baseline characteristics, adjusted comparisons using linear regression methods may be performed. Results will be presented as mean differences, 95% CIs and p values. There will be no adjustment of p values and CIs for multiple comparisons across the seven sub-strata. In addition to analyses within strata, analyses combining all strata will be performed. Analyses will test for different effects of immediate versus deferred vaccination in separate strata by testing for interactions between strata and randomised arm. These results will be presented as forest plots, by strata and overall. Analysis for Group 2 participants will be presented independently using similar approaches as above as appropriate.

Secondary endpoints will include time-weighted AUC change from baseline in anti-tetanus log_10_ NAb titre over 12 months. Analysis will be conducted as described above for the primary endpoint following a mITT approach including all randomised participants with a baseline visit and at least one post baseline time point measured.

A comprehensive statistical analysis plan will be drafted by the study statistician and approved by the PSC prior to data lock. Copies of the analysis plan will be available on request following its finalisation.

#### Methods to handle protocol non-adherence and methods to handle missing data

As indicated above, all participants with a minimum of two study visits will be included in the primary analysis, therefore including participants even with incomplete data at every time point, a strength of using an AUC endpoint. There will be no imputations for missing data in the primary outcome. Secondary analyses will also be conducted in a per-protocol (PP) population including only participants with available data at all time-points and received the scheduled interventions. Protocol non-adherers will also be excluded in the PP analyses.

#### Data safety monitoring committee

The DSMB will comprise at least one biostatistician, one immunologist, one infectious diseases physician and either a haematologist, rheumatologist, gastroenterologist or neurologist. All DSMB members will be independent of the study, the Kirby Institute and UNSW and all agents who fund or support the trial. The DSMB chair will be selected by the members at the first meeting. The study statistician will have an ex-officio role on the DSMB but will not be a voting member.

The Terms of Reference and Operating Guidelines of the DSMB were drafted and finalised in collaboration with DSMB members prior to study commencement.

#### Interim analyses

An interim safety analysis of full summary data examining booster vaccine-associated AEs, vaccine-induced disease flares and breakthrough COVID-19 infections will be performed when 30% (100) of participants reach the 24-week time point. A second safety analysis and an efficacy analysis examining the primary endpoint will be performed when 50% (160) of participants have reached 12 months. Additional meetings at which full interim data summaries will be reviewed may be scheduled following the occurrence of repeated or unresolved clinical events such that the Co-ordinating Principal Investigator (CPI) and DSMB Chair feel a full interim safety summary is warranted, or if the first DSMB meeting indicates a further meeting is warranted prior to all participants completing the study.

DSMB meeting reports will include open and closed session reports. Open session reports will include data on study conduct, protocol compliance, recruitment, baseline and follow-up characteristics and will be distributed to the PSC Chair, study statistician and DSMB members. Outcome results will not be discussed at open sessions. Closed session reports will be provided as unblinded reports and distributed only to DSMB members who will discuss outcome results, make decisions and formulate recommendations.

Following each DSMB review of full safety data, the DSMB will recommend to the PSC through the PI to (a) continue the study without modification, or (b) continue with stated modifications, or c) terminate the study.

### Trial monitoring

Study monitoring will be conducted by routine on-site monitoring visits and by centralised monitoring to confirm the trial is being conducted according to the protocol and regulatory guidelines, to ensure participant safety and to maintain data integrity. Initial on-site monitoring visits will be conducted after five participants have been enrolled or approximately 3 months after recruitment start whichever comes first. Following the initial on-site monitoring visit, subsequent on-site visits will be conducted every 6 months and then at study closeout with centralised monitoring conducted on an ongoing basis during the trial. Sites that enrol a high number of patients over a short period of time (ie, have high recruitment rates), those with higher than expected failure rates, recruitment of ineligible patients, significantly higher or lower per participant AE/SAE rates or where there is an abnormal distribution of critical efficacy and safety data will have on-site monitoring every 6–8 weeks. The sponsor will submit an annual safety report to the central ethics committee for review and consideration.

## Ethics and dissemination

### Research ethics approval

The research protocol and related study documentation were approved by the Western Sydney Local Health District Human Research Ethics Committee on 25 August 2022 (Ref no. 2022/PID00782 – 20022/ETH0069). Following central ethics approval, participating sites sought approval from their local research governance body.

### Protocol amendments

Protocol amendments will also be submitted for approval by the central Human Research Ethics Committee and local research governance bodies. Following ethics approval, investigators will be informed in writing of all changes.

### Dissemination

Study results will be published in peer-reviewed medical journals and presented at local and international conferences. All findings regardless of the outcome will be reported; study data will be shared with other investigators and stakeholders on request. A lay summary of study findings will be prepared in the form of a participant letter and, following ethics approval, will be made available through the clinicians managing participants’ study qualifying medical conditions.

## Supplementary material

10.1136/bmjopen-2025-115259 online supplemental file 1
