# Supplementary material for: Comparison of immunity-boosting regimens for COVID-19 upon initiation of immunosuppressive therapy (CIRCUIT): study protocol for a randomised, controlled clinical trial
Source: BMJ Open. 2026 Apr 24;16(4):e115259. doi: 10.1136/bmjopen-2025-115259 (PMC13110584; doi:10.1136/bmjopen-2025-115259)
Supplement: online supplemental file 1 [file bmjopen-16-4-s001.docx]

**Supplementary Table 1.** Schedules of activities

**a)**

| **Protocol activities** | **Visit 1**  **Screening** | **Visit 2 Randomise**  **/allocation** | **Visit 3**  **Vaccination** | **Visit 4^a^** | **Visit 5** | **Visit 6** | **Visit 7** | **Visit 8** | **Visit 9** | **Visit 10** | **Visit 11** |
| --- | --- | --- | --- | --- | --- | --- | --- | --- | --- | --- | --- |
| Visit weeks and window | Up to 4 weeks pre Week 0 | Up to 2 weeks pre Week 0 | Week  0 | Week 1 ± 2 days | Week 4 ± 7 days | Week 12 ± 7 days | Week 24 ± 7 days | Week 25 ± 2 days | Week 28 ± 7 days | Week 36 ± 7 days | Week 48 ± 7 days |
| Informed consent | X |  |  |  |  |  |  |  |  |  |  |
| Medical history, incl/excl criteria | X |  |  |  |  |  |  |  |  |  |  |
| Vital signs, physical measurements^b^ | X | X |  |  | X | X | X |  | X | X | X |
| Pregnancy test^c^ | X |  |  |  |  |  |  |  |  |  |  |
| Randomisation/allocation |  | X |  |  |  |  |  |  |  |  |  |
| SARS-CoV-2 booster |  |  | X |  |  |  |  |  |  |  |  |
| Immunosuppression start^d^ |  |  | X |  |  |  |  |  |  |  |  |
| DT booster |  |  |  |  |  |  | X |  |  |  |  |
| Adverse events |  |  |  | X |  |  |  | X |  |  |  |
| Concomitant medication |  | X |  |  | X | X | X |  | X | X | X |
| Quality of Life |  | X |  |  |  |  | X |  |  |  | X |
| Blood for serum storage (20 mL) | X | X |  |  | X | X | X |  | X | X | X |
| Blood for PBMC storage (60 mL) |  | X |  |  | X |  | X |  | X |  | X |

**b)**

| **Protocol activities** | **Visit 1**  **Screening** | **Visit 2 Randomise**  **/allocation** | **Visit 3^e^**  **Vaccination** | **Visit 4^a^** | **Visit 5** | **Visit 6** | **Visit 7** | **Visit 8** | **Visit 9** | **Visit 10** | **Visit 11** |
| --- | --- | --- | --- | --- | --- | --- | --- | --- | --- | --- | --- |
| Visit weeks and visit window | Up to 4 weeks pre Week 0 | Up to 2 weeks pre Week 0 | Week  0 | Week 1 ± 2 days | Week 4 ± 7 days | Week 12 ± 7 days | Week 24 ± 7 days | Week 25 ± 7 days | Week 28 ± 7 days | Week 36 ± 7 days | Week 48 ± 7 days |
| Informed consent | X |  |  |  |  |  |  |  |  |  |  |
| Medical history, incl/excl criteria | X |  |  |  |  |  |  |  |  |  |  |
| Vital signs, physical measurements^b^ | X | X |  |  | X | X | X |  | X | X | X |
| Pregnancy test^c^ | X |  |  |  |  |  |  |  |  |  |  |
| Randomisation/allocation |  | X |  |  |  |  |  |  |  |  |  |
| Immunosuppression start^d^ |  |  | X |  |  |  |  |  |  |  |  |
| SARS-CoV-2 booster |  |  |  |  |  |  | X |  |  |  |  |
| DT booster |  |  | X |  |  |  |  |  |  |  |  |
| Adverse events |  |  |  | X |  |  |  | X |  |  |  |
| Concomitant medication |  | X |  |  | X | X | X |  | X | X | X |
| Quality of Life |  | X |  |  |  |  | X |  |  |  | X |
| Blood for serum storage (20 mL) | X | X |  |  | X | X | X |  | X | X | X |
| Blood for PBMC storage (60 mL) |  | X |  |  | X |  | X |  | X |  | X |

**c)**

| **Protocol activities** | **Visit 1**  **Screening** | **Visit 2**  **Randomise**  **/allocation** | **Visit 3 Vaccination** | **Visit 4^a^** | **Visit 5** | **Visit 6** | **Visit 7** | **Visit 8** | **Visit 9** | **Visit 10** |
| --- | --- | --- | --- | --- | --- | --- | --- | --- | --- | --- |
| Visit weeks and visit window | Up to 4 weeks pre Week 0 | Up to 2 weeks pre Week 0 | Week 0 | Week 1  ± 2 days | Week 4  ± 7 days | Week 12 ± 7 days | Week 24 ± 7 days | Week 28 ± 7 days | Week 36 ± 7 days | Week 48 ± 7 days |
| Informed consent | X |  |  |  |  |  |  |  |  |  |
| Medical history, incl/excl criteria | X |  |  |  |  |  |  |  |  |  |
| Vital signs, physical measurements^b^ | X | X |  |  | X | X | X | X | X | X |
| Pregnancy test^c^ | X |  |  |  |  |  |  |  |  |  |
| Randomisation/allocation |  | X |  |  |  |  |  |  |  |  |
| SARS-CoV-2 booster |  |  | X |  |  |  |  |  |  |  |
| Adverse events |  |  |  | X |  |  |  |  |  |  |
| Concomitant medication |  | X |  |  | X | X | X | X | X | X |
| Quality of Life |  | X |  |  |  |  | X |  |  | X |
| Blood for serum storage (20 mL) | X | X |  |  | X | X | X | X | X | X |
| Blood for PBMC storage (60 mL) |  | X |  |  | X |  | X | X |  | X |

**d)**

| **Protocol activities** | **Visit 1**  **Screening** | **Visit 2 Randomise**  **/allocation^e^** | **Visit 3**  **Vaccination** | **Visit 4** | **Visit 5** | **Visit 6** | **Visit 7^a^** | **Visit 8** | **Visit 9** | **Visit 10** |
| --- | --- | --- | --- | --- | --- | --- | --- | --- | --- | --- |
| Visits and visit window | Up to 4 weeks pre Week 0 | Up to 2 weeks pre Week 0 | Week 0 | Week 4  ± 7 days | Week 12 ± 7 days | Week 24 ± 7 days | Week 25 ± 2 days | Week 28 ± 7 days | Week 36 ± 7 days | Week 48 ± 7 days |
| Informed consent | X |  |  |  |  |  |  |  |  |  |
| Medical history, incl/excl criteria | X |  |  |  |  |  |  |  |  |  |
| Vital signs, physical measurements^b^ | X | X |  | X | X | X |  | X | X | X |
| Pregnancy test^c^ | X |  |  |  |  |  |  |  |  |  |
| Randomisation/allocation |  | X |  |  |  |  |  |  |  |  |
| SARS-CoV-2 booster |  |  |  |  |  | X |  |  |  |  |
| Adverse events |  |  |  |  |  |  | X |  |  |  |
| Concomitant medication |  | X |  | X | X | X |  | X | X | X |
| Quality of Life |  | X |  |  |  | X |  |  |  | X |
| Blood for serum storage (20 mL) | X | X |  | X | X | X |  | X | X | X |
| Blood for PBMC storage (60 mL) |  | X |  | X |  | X |  | X |  | X |

**a)** Group 1- arm A, immediate booster; **b)** Group 1 - arm B, deferred booster; **c)** Group 2 - arm C, immediate booster; **d)** Group 2 - arm D, deferred booster

Notes: ^a^Conducted via telephone; ^b^Physical measurements at screening only; ^c^Women of child-bearing potential only; ^d^Immunosuppression should commence 1-14 days post-booster; ^e^Visit 2 (randomise/allocation) and visit 3 (Week 0) can be combined; mL: millilitre; PBMCs: peripheral blood mononuclear cells

**CIRCUIT STUDY**

**Master Participant Information and Consent Forms**

**1. Group 1 Participant Information and Consent Form:**

**2. Group 2 Participant Information and Consent Form:**
