# Supplementary material for: Comparison of immunity-boosting regimens for COVID-19 upon initiation of immunosuppressive therapy (CIRCUIT): study protocol for a randomised, controlled clinical trial
Source: BMJ Open. 2026 Apr 24;16(4):e115259. doi: 10.1136/bmjopen-2025-115259 (PMC13110584; doi:10.1136/bmjopen-2025-115259)
Supplement: Supplementary file 2 [file bmjopen-2025-115259.reviewer_comments.pdf]

PEER REVIEW HISTORY

BMJ Open publishes all reviews undertaken for accepted manuscripts. Reviewers are asked to complete a checklist review form and are provided with free text boxes to elaborate on their assessment. These free text comments are reproduced below.

ARTICLE DETAILS

Title (Provisional)

Comparison of Immunity-boosting Regimens for COVID-19 upon Initiation of immunosuppressive Therapy (CIRCUIT): study protocol for a randomised, controlled clinical trial

Authors

Carey, Dianne L; Ahlenstiel, Golo; Brilot, Fabienne; Brown, David A; Bull, Rowena; Crowther, Helen; Cunningham, Anthony; Davenport, Miles P.; Diamond, Peter; Hamad, Nada; Johnston, Amanda; Kelleher, Anthony D; Lee, Frederick J; Matthews, Gail V.; Petoumenos, Kathy; Quichua, Gesalit C; Swaminathan, Sanjay; Trotman, Judith; Turville, Stuart; Vucic, Steve; Wong, Peter; Sasson, Sarah

---

VERSION 1 - REVIEW

**Reviewer**	**1**
**Name**	**Pickering, Harry**
**Affiliation**	**London School of Hygiene & Tropical Medicine**
**Date**	**27-Jan-2026**
**COI**	**None**

Abstract, intro

- Might be clearer as “prolonged…viral infection” or “delayed…viral clearance”

- What about solid organ transplant as well as BMT?

- Would be clearer to say “week 24 post-start of immunosuppression”, or something similar. Prior to reading the methods part it wasn’t clear what week 24 was referring to

Intro

- The sentences at the end of page 12 are about vaccine responses by type of immunosuppression (“However, post COVID-19 vaccination…”), unless this study is investigating this, which I don’t believe it is, I would cut

- The intro covers responses to mostly two-dose series of the vaccination, but the study is about boosting, would add literature around the impact of boosting in these populations

- Is there a particular rationale for week 24?

Methods

- When discussing tixagevimab/cilgavimab on page 21, how will the assays be adapted to distinguish natural immunity vs immune responses to the treatment?

- Some basic information, or a reference, for the neutralization assay would be beneficial

- In regards to participant timeline, is there a minimum number of completed visits to be included in the final analysis?

- On page 23, given the described differences in immune response to vaccination, probably not ideal to have calculate required sample size using estimates from healthy individuals

- On page 27, is one post-baseline timepoint sufficient to calculate the time-weighted AUC for NAb? And can it be any timepoint? I would be concerned about using only one timepoint, particularly if it was early post-booster

- On page 29, the statement about more frequent sites visits is quite vague. What does more frequent mean? What does higher recruitment rates mean? Would recruitment of one ineligible patient lead to increased visits?

VERSION 1 - AUTHOR RESPONSE

Reviewer #1 Report

Dr Harry Pickering

London School of Hygiene and Tropical Medicine

Comments to the Author:

Abstract, intro

1. **Might be clearer as "prolonged…viral infection" or "delayed…viral clearance"**

   We thank the reviewer for this suggestion. The first sentence of the Abstract has now been changed to 'Immunosuppression is associated with an increased risk of delayed SARS-CoV-2 viral clearance, severe COVID-19 and related death'.

2. **What about solid organ transplant as well as BMT?**

We thank the reviewer for this comment. This research is funded by the Australian Government Ministry of Health's Medical Research Future Fund (MRFF). In the same grant round another RCT entitled BOOST-IC was funded which was designed to enrol adults with specific haematological malignancies (chronic lymphocytic leukaemia, non-Hodgkin's lymphoma and multiple myeloma), HIV infection and solid organ transplant recipients. In the interest of both studies not competing for the same patient groups, we decided not to include people with HIV infection and solid organ transplant recipients in CIRCUIT. The following explanatory information has been added to page 11 of the manuscript 'Due to a related study running concurrently in Australia[20] people with HIV infection and solid organ transplant recipients were not included in the CIRCUIT Study'.

3. **Would be clearer to say "week 24 post-start of immunosuppression", or something similar. Prior to reading the methods part it wasn't clear what week 24 was referring to**

   We thank the reviewer for this suggestion. We have now amended this section of the manuscript to read '24 weeks after new or intensified immunosuppression i.e. Week 24 timepoint'.

Introduction

4. **The sentences at the end of page 12 are about vaccine responses by type of immunosuppression ("However, post COVID-19 vaccination…"), unless this study is investigating this, which I don't believe it is, I would cut.**

   We thank the reviewer for this suggestion. However, although the primary aim of the research is to investigate the timing of a COVID-19 booster (i.e. given at Week 0 versus Week 24), we are also interested in examining possible differences in vaccine response between the various treatment types in the Group 1 Autoimmunity/

Inflammatory subgroup, which is the reason for the subdivision into treatment type (see Table 1). As outlined in the Strengths and Limitations section, 'The study has statistical power to analyze Group 1 participants as a whole or by distinct substrata-based type of immunosuppression'. Therefore, it is our strong preference not to amend this section of the manuscript.

5. **The intro covers responses to mostly two-dose series of the vaccination, but the study is about boosting, would add literature around the impact of boosting in these populations.**

   We thank the reviewer for this comment. In response, we have added on pages 8 and 9 details of a number of landmark studies that investigated seroconversion to a third dose of a COVID-19 vaccine in the setting of a haematological malignancy or an autoimmune/inflammatory disease.

6. **Is there a particular rationale for week 24?**

   We thank the reviewer for this question. The Week 24 timepoint was chosen for two reasons: 1) most patients with a haematological malignancy or an autoimmune/inflammatory disorder undergo an "induction phase" of more intense immunosuppression at the start of treatment. Often by 24 weeks (~six months) the immunosuppression can be "stepped down" to a lower maintenance regimen. Hence, the Week 24 timepoint was deemed a suitable time to administer a COVID-19 booster to compare with the pre-immunosuppression administration timepoint. Additionally, 2) from a pragmatic point of view, the study design allows for comparison between a COVID-19 booster vaccine given at either Week 0 or Week 24. The study follows participants for a total of 48 weeks. While booster administration timepoints after week 24 were considered for participants in Group 1 Arm 2 (i.e. deferred COVID-19

booster arm) such as Week 36 which would require a longer follow-up period increasing the risk of losses to follow-up.

Methods

7. **When discussing tixagevimab/cilgavimab on page 21, how will the assays be adapted to distinguish natural immunity vs immune responses to the treatment?**

   We thank the reviewer for this important question. The synthetic monoclonal antibodies tixagevimab/cilgavimab (Evusheld) have been modified to not bind to Fc gamma receptors (FcγRs). The FcγR is an endogenous receptor that binds naturally occurring antibodies produced by the host. The modifications made to tixagevimab/cilgavimab were aimed at reducing antibody-dependent cellular cytotoxicity (ADCC) and related inflammation. Therefore, participants who receive tixagevimab/cilgavimab, including an FcγR binding step in the antibody detection system will selectively identify host-produced anti-spike IgG and not the synthetic antibodies of Evusheld. To clarify matters, we have now included this information in the manuscript on page 18.

8. **Some basic information, or a reference, for the neutralization assay would be beneficial.**

   We thank the review for this helpful suggestion. The SARS-CoV-2 neutralizing antibody assay that will be utilised is a high-throughput platform assay developed by our group. The assay involves culturing a cell-line hyper-permissible to SARS-CoV-2 due to the expression of ACE2 and TMPRSS2 receptors. Cell culture wells are then incubated with serial dilution of patient serum as well as homogenous concentrations of SARS-CoV-2 virions including variants of concern. Anti-spike IgG present in the patient serum acts to block viral entry into the cell. The assay readout is the infectious dose 50% (ID_{50}) which is the viral concentration required to obtain 50% cellular

death. We have referred to the assay in the Abstract and included its details and appropriate references in the Outcomes section (16) of the manuscript (page 18).

9. **In regards to participant timeline, is there a minimum number of completed visits to be included in the final analysis?**

   We thank the reviewer for this question. As outlined on pages 24/25 of the manuscript 'The primary analysis will follow a modified intention to treat approach (mITT)and include all randomised participants with a baseline visit and at least one post baseline time point measured. This approach allows for the inclusion of all participants, even those with partial follow-up'.

10. **On page 23, given the described differences in immune response to vaccination, probably not ideal to have calculate required sample size using estimates from healthy individuals**.

    We thank the reviewer for this comment. We agree it would have been preferable to use vaccination responses in immunocompromised individuals for our original power calculations. However, this research project was designed in 2021 when there were little available data on COVID-19 vaccine responses in immunocompromised individuals as they were excluded from the original vaccine licencing trials.

11. **On page 27, is one post-baseline timepoint sufficient to calculate the time-weighted AUC for NAb? And can it be any timepoint? I would be concerned about using only one timepoint, particularly if it was early post-booster.**

    We thank the reviewer for this important question. As outlined on pages 24/25 of the manuscript, 'The primary analysis will follow a modified intention to treat approach (mITT) and include all randomised participants with a baseline visit and at least one post baseline time point measured. This approach allows for the inclusion of all participants, even with partial follow-up. The AUC will be calculated for each

participant using the trapezoidal rule based on all available data'. We acknowledge the real-world challenges that on-study deaths, losses to follow-up and study withdrawals present for a project where the primary endpoint requires multiple longitudinal follow-up timepoints. We strongly support an analysis that follows a mITT approach as per the original protocol. However, we acknowledge the reviewer's concerns and agree it will be important to monitor the proportion of participants with missing data across the study arms. In addition to the mITT analysis, we will perform an additional secondary analysis which will only including participants with complete data and these findings will be compared to the mITT analysis. Additionally, if the proportion of participants with missing data is higher in one study arm, additional sensitivity analysis will be performed. Details of these additional analyses have been inserted on page 25 of the manuscript.

12. **On page 29, the statement about more frequent sites visits is quite vague. What does more frequent mean? What does higher recruitment rates mean? Would recruitment of one ineligible patient lead to increased visits?**

    We thank the reviewer for these questions. Following the initial on-site monitoring visit, subsequent on-site visits will be conducted every six months and then at study closeout with centralised monitoring conducted on an ongoing basis during the trial. Sites that enrol a high number of patients over a short period of time (i.e. have high recruitment rates) will have on-site monitoring every six to eight weeks. Recruitment of one ineligible patient in isolation would be unlikely to increase the frequency of visits unless it was combined with other issues of concern such as protocol or regulatory non-compliance, concerns for participant safety following review of adverse event reports or data integrity problems. This information has now been included on page 27 of the manuscript.

VERSION 2 - REVIEW

**Reviewer**	**1**
**Name**	**Pickering, Harry**
**Affiliation**	**London School of Hygiene & Tropical Medicine**
**Date**	**16-Mar-2026**
**COI**	

Thanks for addressing my prior comments with some key added details here, no additional comments from me.
